# Monte Carlo Characterization of the Trimage Brain PET System

**DOI:** 10.3390/jimaging8020021

**Published:** 2022-01-23

**Authors:** Luigi Masturzo, Pietro Carra, Paola Anna Erba, Matteo Morrocchi, Alessandro Pilleri, Giancarlo Sportelli, Nicola Belcari

**Affiliations:** 1 Department of Physics “E. Fermi”, University of Pisa, 56127 Pisa, Italy; l.masturzo@studenti.unipi.it (L.M.); pietro.carra@df.unipi.it (P.C.); matteo.morrocchi@pi.infn.it (M.M.); alessandro.pilleri@df.unipi.it (A.P.); nicola.belcari@unipi.it (N.B.); 2 National Institute of Nuclear Physics (INFN), Pisa Section, 56127 Pisa, Italy; 3 Department of Translational Research and New Technology in Medicine and Surgery, Regional Center of Nuclear Medicine, Azienda Ospedaliero Universitaria Pisana, University of Pisa, 56126 Pisa, Italy; paola.erba@unipi.it

**Keywords:** brain PET, Monte Carlo characterization, PET/MR, TRIMAGE project, NEMA

## Abstract

The TRIMAGE project aims to develop a brain-dedicated PET/MR/EEG (Positron Emission Tomography/Magnetic Resonance/Electroencephalogram) system that is able to perform simultaneous PET, MR and EEG acquisitions. The PET component consists of a full ring with 18 sectors. Each sector includes three square detector modules based on dual sstaggered LYSO:Ce matrices read out by SiPMs. Using Monte Carlo simulations and following NEMA (National Electrical Manufacturers Association) guidelines, image quality procedures have been applied to evaluate the performance of the PET component of the system. The performance are reported in terms of spatial resolution, uniformity, recovery coefficient, spill over ratio, noise equivalent count rate (NECR) and scatter fraction. The results show that the TRIMAGE system is at the top of the current brain PET technologies.

## 1. Introduction

Over the past 40 years, Positron Emission Tomography (PET) brain imaging has allowed unique insights into brain function under normal conditions and in disease states [1]. The ability of PET to provide spatial localization of metabolic changes and to accurately and consistently quantify their distribution proved valuable for applications in brain imaging. The first fundamental part of a PET study is the radiotracer: following the biological path of this chemical compound, it is possible to observe specific phenomena in the brain. In all neurodegenerative diseases, damage to neuronal function and therefore reduced energy metabolism occur. Fluorodeoxyglucose (${}_{}^{18}F$-FDG) can be used to detect this impairment and it is well known that different diseases show distinct patterns of reduced ${}_{}^{18}F$-FDG uptake. Many neurological disorders are often difficult to distinguish, thus developing molecular imaging approaches that aim at identifying such pathologies as well as supporting development of modifying therapies is a very active area of research. Although ${}_{}^{18}F$-FDG is the most widely used radiopharmaceutical, it is not the ideal tracer for brain imaging, owing to its high physiological cortical uptake and lack of specificity. This has opened the way for the introduction of several novel radiotracers, each with their own inherent strengths and limitations [2,3] that can increase the potential of brain imaging. A specific discussion of the state of the art of PET radiotracers is beyond the scope of this work; however, we refer the reader to [4,5,6] for more in-depth information about this topic. The present and future availability of the new radiotracers have raised a new interest in brain-dedicated PET systems, especially if combined with magnetic resonance (MR), having superior performance than whole body systems.

Multimodal imaging, specifically PET/CT, brought a new perspective into the fields of clinical and preclinical imaging as the combination of anatomical structures, revealed from CT, and the functional information from PET are fused into one image: with high fusion accuracy it can provide an advanced diagnostic tool and research platform. Although PET/CT is already an established clinical tool, it still bears some limitations. A major drawback is that CT provides only limited soft tissue contrast and exposes the subject to a significant radiation dose. Recent research concentrates on the combination of PET and MR into one single machine [7]. The goal of this development is to integrate the PET detectors into the MRI scanner which would allow simultaneous data acquisition, resulting in combined functional and morphological images with an excellent soft tissue contrast, very good spatial resolution of the anatomy and very accurate temporal and spatial image fusion. Additionally, since magnetic resonance imaging (MRI) also provides functional information [8] such as blood oxygenation level-dependent imaging (which measures the proportion of oxygenated haemoglobin in specific areas of the brain mirroring blood flow) or spectroscopy (for measuring bio-chemical changes in the brain), PET/MRI could even provide multi-functional information of physiological processes in vivo. Although MRI measures signals based on proton density and is unable to provide an analogous attenuation map compared to CT, many MRI-based attenuation corrections have been pursued such as, for example, atlas algorithms and direct MRI imaging [9]. A complete review of attenuation correction methods is beyond the scope of this paper: we refer the reader to [10,11,12,13,14] for more information about this topic.

Some of the factors that degrade PET spatial resolution may be optimized to the specific case of brain imaging. The object of study being the head, the diameter of the scanner can be reduced with respect to a whole body PET, which implies a lower contribution of the non-collinearity effect to the spatial resolution. Reducing the diameter of the scanner in combination with increasing the area of the detector increases the solid angle coverage and, thus, the sensitivity. Started at the end of 2013 and held by an international consortium, TRIMAGE is a project with the aim of creating a trimodal, cost-effective imaging tool consisting of PET/MR/EEG (Electroencephalogram). The target applications of the TRIMAGE PET/MRI system are mainfold. Exploiting new radiotracers specially made for PET/MRI imaging could provide better studies on neurodegenerative diseases (Parkinson and Dementia). A precise assessment of the involvement of the Central Nervous System (CNS) in systemic autoimmune diseases could be performed as well as multi-parameter imaging of brain tumors. The system is mainly unique for being a brain PET/MR imager with a cryogen-free MR subsystem. It was also designed to keep compatibility with commercial MR-compatible EEG systems [15,16]. EEG is advantageous for its temporal resolution, far better than in other imaging methods and for the time course analysis. On the other hand, it suffers from relatively poor spatial information. The full integration of these three different diagnostic modalities could provide complementary anatomical, physiological, metabolic and functional information about the brain.

In this work, Monte Carlo simulations have been performed using the results obtained in the previous system characterization [17] in order to estimate the image quality of the scanner. The final results will be used as experimental validation for real data. In Table 1 and Table 2, the features of other PET and PET/MR systems found in the literature are listed. The Full Width Half Maximum (FWHM) and sensitivity values are referred to the center of the Field Of View (FOV). In Table 3, the NEMA standards used to evaluate the performance of the systems are reported.

## 2. Materials and Methods

### 2.1. System Description

**Figure 1:**
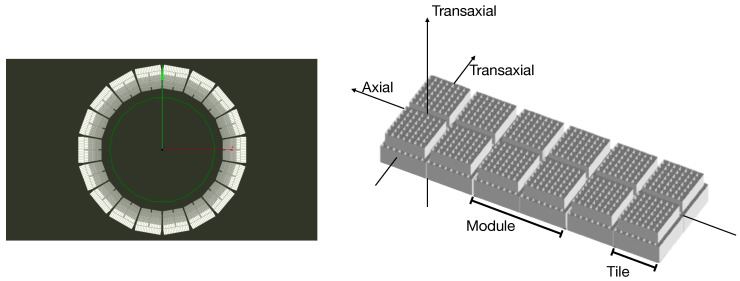
(**Left**) Simulated view of TRIMAGE full detector ring. (**Right**) Schematic view of a sector.

The TRIMAGE PET scanner consists of 18 sectors, each one composed of 3 square detector modules. Each module includes 4 submodules called tiles. In Figure 1, a view of the system is reported.

**Figure 2:**
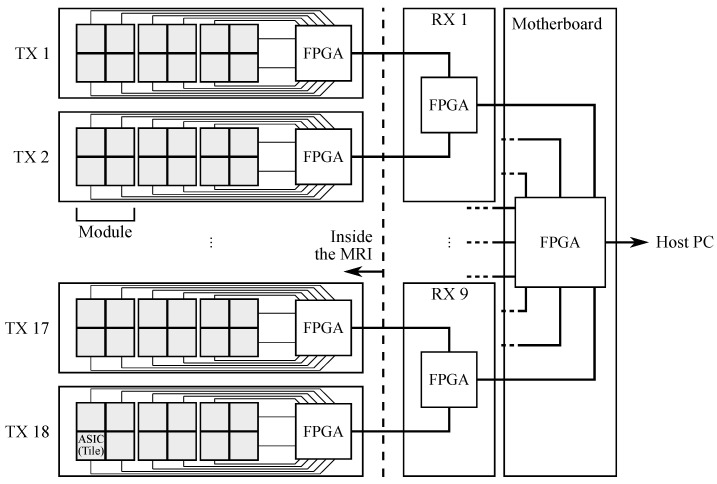
Schematic diagram of the acquisition pipeline from the ASICs to the host PC.

Each tile features two segmented LYSO:Ce crystal layers. The top layer consists of 7 × 7 crystals of 3.3 × 3.3 × 8 mm${}^{3}$, while the bottom layer, centered with respect to the top layer, consists of 8 × 8 crystals of 3.3 × 3.3 × 12 mm${}^{3}$. The crystals in both layers have a pitch of 3.4 mm and both layers are staggered by a half-pitch. This configuration permits both reducing the depth of interaction (DOI) uncertainty and achieving a better sampling of the FOV with respect to a single layer with the same pixel pitch. A black thin separator is placed between adjacent bottom layers to reduce the optical crosstalk between tiles. An enhanced specular reflector (3M ESR) is placed on the lateral sides of each crystal, while the open face of the top layer is covered with a white Teflon tape. There are 216 tiles in the whole system, corresponding to 24,408 (216 × 113) crystals. The AFOV and TFOV are, respectively, 164 and 260 mm. The crystals in the bottom layer are directly coupled to 64 near ultraviolet SiPMs that are arranged in two matrices, specifically designed and manufactured by AdvanSiD s.r.l., Trento, Italy. Each element has a size of 3 × 3 mm${}^{2}$ and a pitch of 3.4 mm in order to match the scintillator pitch. Each SiPM has 5520 micro-cells, 40 μm side with a 60% fill-factor. The 64 signals from a tile are read out by a 64-channel TRIROC ASIC [38]. Four TRIROC ASICs are hosted on a front-end board which we refer to as the ASIC board. The ASIC board can read out all 256 output signals from a module and the complete PET front-end data acquisition system is composed by 54 ASIC boards. The digital part of the ASIC manages the conversion and the data transmission to the front-end FPGA-based board (called the TX board), which computes the timestamp associated to each triggered SiPM and transmits the acquired data to the back-end for coincidence processing [39]. A data packet corresponds to every single event that is stored in FPGA for online processing: here, the interaction position, the final timestamp and the whole energy released are computed. The back-end system is composed of a motherboard (MB) and 9 receiver boards (called RX boards). Each RX board receives data from two TX boards. A schematic diagram of the acquisition pipeline is shown in Figure 2, while a more in-depth review of the data acquisition system can be found in [39].

The coincidence window is 5 ns. Random coincidence rates are determined with the delayed window technique. The image reconstruction process is implemented using an in-house-developed software. The system matrix *S*, that models the imaging system in reconstruction, is factorized into the following components:

- N (Normalization)—a diagonal matrix containing the normalization coefficient for every line of response;
- A (Attenuation)—a matrix containing the attenuation coefficient for every line of response;
- R (Blurring)—a matrix that models the blurring in the object space;
- G (Geometry)—a matrix that maps the link between the object space and the projection space.

The reconstruction software performs Maximum Likelihood Expectation Maximization (MLEM) with an image-space modelling of the spatial resolution [40,41,42]. This is performed using a space-invariant 3D gaussian kernel (FWHM = 2.3 mm) to model the PSF of the acquisition system. Regularization techniques are mathematical algorithms used to reduce noise and improve image quality. The regularization technique proposed by Wang and Qi [43] has been implemented in the reconstruction process.

### 2.2. Performance Evaluation Procedure

**Figure 3:**
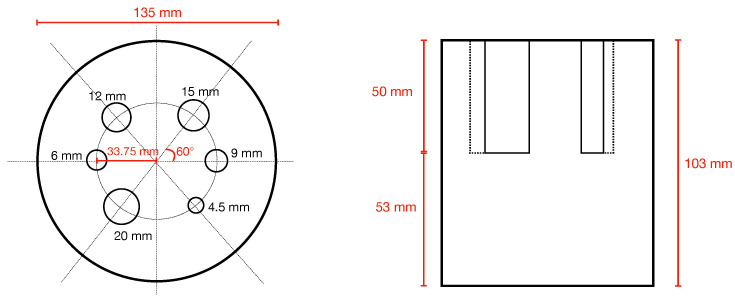
Top and transverse view of simulated phantom. Near each rod the diameter of the rod is indicated (in black).

At the time of writing, specific NEMA procedures for brain PET imaging do not exist. Possible alternatives are the NEMA NU2-2012 [44], describing whole body PET performance measurement procedures and the NEMA NU4-2008 [45], dedicated to small-animal PET performance evaluations. However, none of them are fully applicable to brain PET, in particular regarding the estimation of image quality performance. In fact, NU2-2012 uses a torso-like phantom whose dimensions do not fit the TRIMAGE FOV, while NU4-2008 uses a phantom with rods too little to be visible in a brain scanner. Following the idea proposed by Moliner L. et al. [24], we have applied a method using a rod phantom similar to the one described in NU4-2008 but with a larger size, better mimicking a human head and following procedure for image quality measurements as described in NEMA NU4-2008. The phantom is 103 mm in height and has a diameter of 135 mm. In the upper half, 6 rods with a height of 50 mm and a diameter of 20, 15, 12, 9, 6 and 4.5 mm are circularly placed (see Figure 3).

The whole phantom, as well as the rods, can be filled with different activity and, in this study, the ratio between the activity concentration in the rods and the activity concentration of the whole phantom was 4:1. To evaluate the ability of reconstruction of cold rods, in the two biggest rods, no activity was simulated: one was filled with non-radioactive water while the other one was filled with air. The whole scanner has been simulated using the GATE software [46] and the input parameters to the simulations, such as energy resolution (17.8% ± 0.4), coincidence window (5 ns) and dead time (17.2 μs), have been experimentally measured as described in the paper reporting the detector performance [17]. Although the materials used for optical separation among crystals and layers have not been directly included in the simulation, the aforementioned input parameters are evaluated in the presence of optical materials. In general, NEMA NU4-2008 guidelines appear to be more applicable to a small-medium FOV PET such as TRIMAGE. We have followed these procedures when possible, but applying several adaptations when needed (see Table 4). In detail, the following figures of merit have been found:

Sensitivity. A ${}_{}^{22}\mathrm{Na}$ spherical source (radius = 0.1 mm) embedded in an acrylic cube (length side = 10 mm) has been used. The activity was 500 kBq in order to avoid dead-time effects. Two energy windows were considered: 250 to 750 keV and 350 to 650 keV.

Spatial resolution. In order to use the same reconstruction algorithm for the whole system characterization, a series of point sources in a warm background were simulated [47]. The necessity of having a warm uniform background is dictated by the non-linearity of the reconstruction algorithm and its non-negativity constraint: if not added, the spatial resolution is underestimated. The radial location of the sources were, starting from the center, 0, 5, 10, 15, 25, 50, 75 and 100 mm. The same procedure was repeated in the transversal plane that stands at 1/4 of the axial FOV. In addition, a Derenzo phantom with 6 groups of rods was simulated. The radius of the rods ranged from 1.8 mm to 4.3 mm in steps of 0.5 mm. The activity was 5.3 kBq/ml of ${}_{}^{18}F$ and the acquisition time was 300 s.

Image Quality. The uniform region of the image quality phantom has been filled with 5.3 kBq/ml of ${}_{}^{18}F$ and the acquisition time was 1200 s. The uniformity is computed as the ratio of standard deviation to the mean of a volume of interest (VOI) taken in the bottom part of the phantom (the uniform region). The VOI was a cylinder with a diameter of 101 mm (75% of the phantom diameter) and a height of 10 mm. To evaluate the ability to discern hot and cold regions, Recovery Coefficient (RC) and Spill Over Ratio (SOR) have been computed and the real activity was estimated as the mean of the uniform region. The exact procedures to calculate the activity in both hot (for RC) and cold rods (for SOR) are reported in [45].

NECR and SF. The scattered events can be expressed as the ratio of scattered events to the sum of scattered and true events: this quantity is known as Scatter Fraction (SF). Two phantoms were used to evaluate both NECR and SF. The first one is the rat-like phantom (described in NU4-2008 protocol [45]), while the second one was a polyethylene cylinder with a diameter of 20 cm and a height of 15 cm. We refer to this as a head-like phantom. The line ${}_{}^{18}F$ source was placed 4.5 cm from the axial center.

## 3. Results

**Figure 4:**
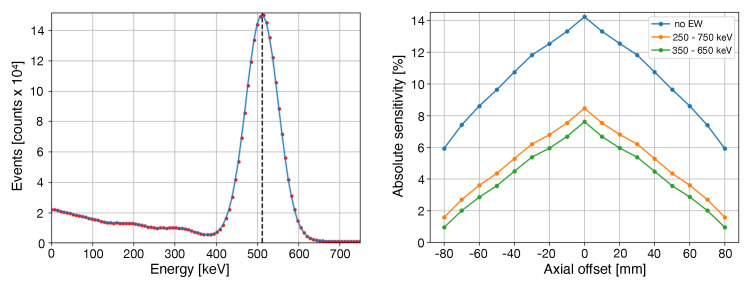
(**Left**) Coincidence energy spectrum. (**Right**) Sensitivity values across the axial direction for different energy windows.

Table 5 reports the sensitivity values found for different energy windows while, in Figure 4, the sensitivity values along the axial direction as well as the coincidence energy spectrum are reported.

**Figure 5:**
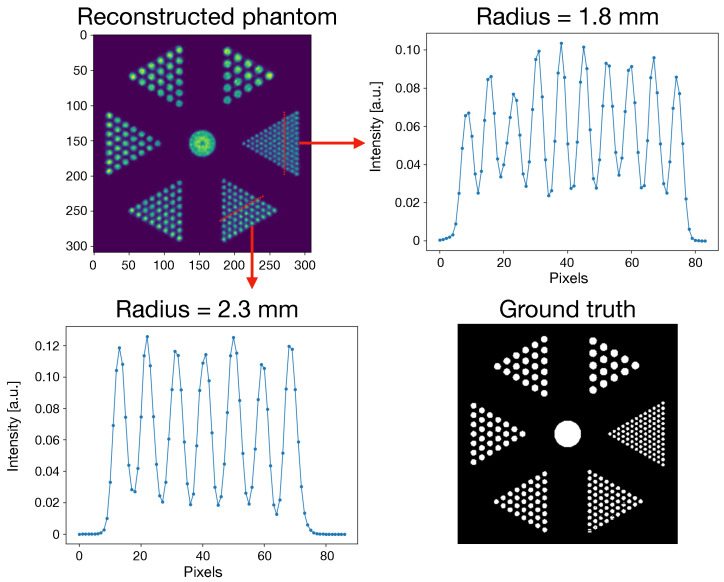
Reconstructed Derenzo Phantom at the 100th iteration number. (**Upper Left**) Reconstructed slice of the phantom. (**Upper Right**) Line profile of the 1.8 mm rods. (**Lower Left**) Line profile of the 2.3 mm rods. (**Lower Right**) Phantom ground truth.

The values of axial and transverse spatial resolution for all the positions discussed in Section 2.2 are reported in Table 6, while the reconstructed image of the Derenzo phantom and the line profiles of the two smallest groups of rods are reported in Figure 5. For spatial resolution, as well as for the other measurements, the MLEM iteration to which the results are referred to is the 100th one.

Table 7 reports the values of uniformity at different iteration number.

**Figure 6:**
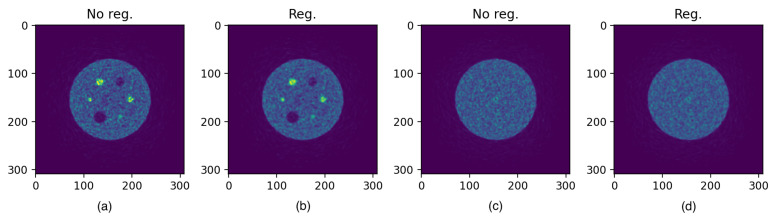
Reconstructed images at the 100th iteration. (**a**,**c**) are the non regularized images, while (**b**,**d**) are the regularized images.

The values of RC and SOR are reported in Table 8. To give an idea of the final output of reconstruction, in Figure 6, the reconstructed images (both regularized and non regularized) of the image quality phantom are presented.

**Figure 7:**
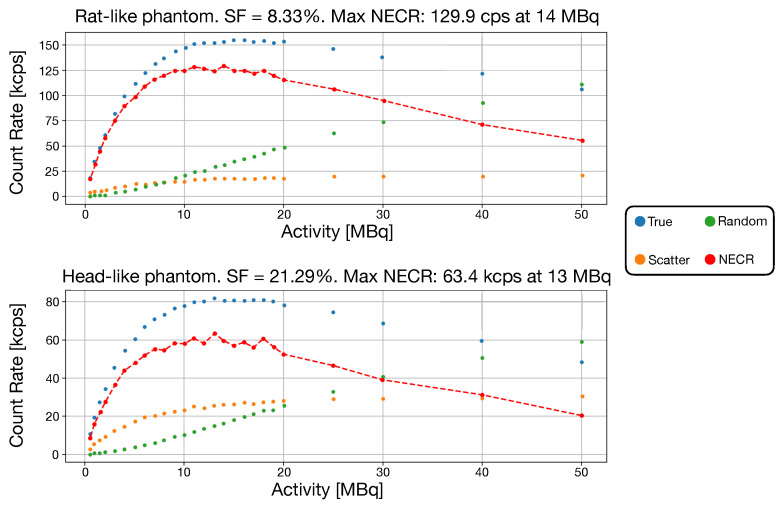
In both images, the NECR data, true, scatter and random events are reported. A red-dashed line is traced between NECR points. (**Top**) NECR for the rat-like phantom. (**Bottom**) NECR for the head-like phantom.

The two NECR curves and the relative scatter fraction, respectively, for the rat-like phantom and head-like phantom, are reported in Figure 7. Both scatter and random coincidences are found directly from simulation.

## 4. Discussion

In this paper, we showed the results of the brain PET component developed for TRIMAGE. The system showed a physical sensitivity at the center of the FOV (CFOV) with a small size ${}_{}^{22}\mathrm{Na}$ source of 7.61% for an energy window of 350–650 keV. A direct comparison can be done with the CareMiBrain (7%), MINDview (7%) and Won et al. scanner (6.9%), as they evaluated the sensitivity following the same standard used in this paper (and almost with the same energy window).

Exploiting the peculiar staggered crystal configuration, it is possible to reach a spatial resolution of 1.9 and 2.25 mm (at the CFOV, axial and transversal, respectively). Moving away from the CFOV, both values deteriorate (4.6% transversely and 5% axially). This worsening can be explained considering that the density of LORs is maximum at the CFOV, and decreasing elsewhere. All the groups of rods of the Derenzo phantom were successfully identified and reconstructed.

We also evaluated the imaging performance of the system. As expected, noise increases with the iteration number: the iterative algorithm, while it converges to the solution, tends to raise the noise in the whole image. This behaviour results in a noisy image (see Figure 6) which is improved by the use of a regularizing technique.

RC and SOR values are close to their theoretical values (1 and 0, respectively) meaning that the capability and quality of reconstruction is well preserved for all the rods. A direct comparison (as it is the only system that uses the same quality phantom described in this work) can be done with the CareMiBrain system: both RC and SOR values are slightly better with respect to the TRIMAGE counterparts. The reason for this mismatch could probably be sought both in the different reconstruction method used and in the application of the mentioned regularization algorithm.

The NECR curve peak results are 129.9 kcps at 14 MBq for the rat-like phantom and 63.4 kcps at 13 MBq for the head-like phantom. The system showed a SF of 8.33% and 21.29% for the rat and head-like phantom, respectively. Given that a specific NEMA procedure for brain imaging does not exist, the scatter phantom used in the state of the art systems are different, therefore a direct comparison cannot be done. Considering the head-like phantom, the prompt events rate (accounting also for the delayed coincidences) in TRIMAGE is about 132 KHz. The size of a single coincidence is 24 bytes; thus, the data transfer rate is 3.1 MB/s: this value is far from the maximum transfer data speed (20 MB/s), meaning that there is no event loss during acquisition.

## 5. Conclusions

In this work, we characterized the PET component of the TRIMAGE brain scanner. Monte Carlo simulations were configured with the detector experimental parameters and have been used to perform the system characterization based on NEMA protocols. NEMA standards are extremely useful in defining system performance and for comparing different devices. It may be useful, in the future, to define a specific standard for brain scanners. Our study shows that the scanner has achieved a good combination of performance in terms of spatial resolution, sensitivity, scatter fraction and image quality.

## Figures and Tables

**Table 1:** Main specifications of brain PET state of the art systems. The Transaxial Field Of View (TFOV) and Axial Field Of View (AFOV) are reported as well as spatial resolution (in terms of Full Width Half Maximum), sensitivity and the energy window (EW) applied.

	TFOV [mm]	AFOV [mm]	FWHM [mm]	Sens.	EW [keV]
HRRT [18]	312	252	≃2.5	4.3%	350–650
jPET-D4 [19,20,21]	390	260	3	9.82%	400–600
Neuro PET/CT [22]	357	220	3	0.75%	400–650
PET-Hat [23]	280	N.A.	4.2	0.72%	>350
CareMiBrain [24]	256	154	≃1.7	7%	355–664
Hamamatsu [25]	330	201.6	2	2.14%	400–650

**Table 2:** Main specifications of brain PET/MR state of the art systems. The TFOV and AFOV are reported as well as spatial resolution, sensitivity and the EW applied.

	TFOV [mm]	AFOV [mm]	FWHM [mm]	Sens.	EW [keV]
BrainPET [26]	320	191	≃3	≃7%	420–600
MINDview [27,28]	240	160	≃1.7	7%	350–650
Jung et al. [29]	390	60	3	0.8%	350–650
Nishikido et al. [30,31,32]	247.8	12	2.3	N.A.	N.A.
Won et al. [33]	256	167	≃2.5	6.9%	350–650

**Table 3:** NEMA standards (spatial resolution, sensitivity, image quality and noise equivalent count rate (NECR) ) used for evaluating system performance. The asterisk means that modifications have been done to the original standard.

	Spatial Resolution	Sensitivity	Image Quality	NECR
HRRT	none	none	none	NEMA 1991 [34]
jPET-D4	NU2-2001 [35]	none	none	NU2-2001
Neuro PET/CT	NU2-2012	NU2-2012	none	NU2-2012
PET-Hat	none	none	none	NU2-2001
CareMiBrain	NU4-2008	NU4-2008	NU4-2008	NU2-2012
	NU2-2012	NU2-2012		
Hamamatsu	NU4-2008	NU2-2012	none	NU2-2012
BrainPET	NU2-2007 [36] *	NU2-2007	NU2-2007 *	NU2-2007
MINDview	NU4-2008 *	NU4-2008	NU4-2008	none
Jung et al.	none	none	none	none
Nishikido et al.	none	none	none	none
Won et al.	NU4-2008 */	NU4-2008 */	none	none
	NU2-2018 [37]	NU2-2018		

**Table 4:** NEMA NU4-2008 measurements performed and the main differences adopted for this study.

Measurements	NU4-2008	Differences
Spatial	Encapsulated ${}_{}^{22}\mathrm{Na}$ source reconstructed	MLEM algorithm instead FBP
resolution	with FBP	
Sensitivity	Encapsulated ${}_{}^{22}\mathrm{Na}$ source reconstructed	MLEM algorithm instead FBP
	with FBP	
Image	Customized phantom with uniformity	Different phantom
Quality	region, rods (hot/cold), (air/water).	structure
phantom	Filled with ${}_{}^{18}F$	
Scatter	Cylindrical polyethylene	Head-like phantom
fraction	phantom. Mouse, rat	
	and monkey dimensions	

**Table 5:** Sensitivity results for different energy windows.

No Energy Window	250–750 keV	350–650 keV
14.22%	8.46%	7.61%

**Table 6:** Values of the spatial resolution at the axial center and at 1/4 of the axial center. All values are in mm.

At Axial Center	0	5	10	15	25	50	75	100
Transverse	2.25	2.275	2.285	2.305	2.305	2.31	2.345	2.355
Axial	1.9	1.92	1.92	1.94	1.96	1.96	2	2.1
**At 1/4 Axial Center**	**0**	**5**	**10**	**15**	**25**	**50**	**75**	**100**
Transverse	2.275	2.285	2.3	2.32	2.335	2.385	2.425	2.44
Axial	1.9	1.9	1.92	1.93	1.97	2.1	2.1	2.3

**Table 7:** Values of Uniformity at different MLEM iterations. All values are in %.

10	20	30	40	50	60	70	80	90	100
4.19	7.01	9.58	11.82	13.96	15.84	17.55	19.13	20.56	21.88

**Table 8:** RC and SOR values for different rods. The SOR is calculated on bigger cold rods (20 and 15 mm), while RC is calculate on the remaining hot rods.

SOR	SOR	RC	RC	RC	RC
**20 mm**	**15 mm**	**12 mm**	**9 mm**	**6 mm**	**4.5 mm**
0.054 ± 0.002	0.094 ± 0.002	0.94 ± 0.01	0.89 ± 0.01	0.88 ± 0.01	0.82 ± 0.01

## Data Availability

The data presented in this study are available on reasonable request from the corresponding author.

## References

[1] Jones T., Rabiner E.A. (2012). The Development, Past Achievements, and Future Directions of Brain PET. J. Cereb. Blood Flow Metab..

[2] D’Souza M., Sharma R., Tripathi M., Hazari P.P., Jaimini A., Mondal A. (2011). Novel positron emission tomography radiotracers in brain tumor imaging. Indian J. Radiol. Imaging.

[3] Zimmer L., Luxen A. (2012). PET radiotracers for molecular imaging in the brain: Past, present and future. Neuroimage.

[4] McCluskey S.P., Plisson C., Rabiner E.A., Howes O. (2020). Advances in CNS PET: The state-of-the-art for new imaging targets for pathophysiology and drug development. Eur. J. Nucl. Med. Mol. Imaging.

[5] Tiepolt S., Patt M., Aghakhanyan G., Meyer P.M., Hesse S., Barthel H., Sabri O. (2019). Current radiotracers to image neurodegenerative diseases. EJNMMI Radiopharm. Chem..

[6] Borja A.J., Hancin E.C., Raynor W.Y., Ayubcha C., Detchou D.K., Werner T.J., Revheim M.E., Alavi A. (2021). A Critical Review of PET Tracers Used for Brain Tumor Imaging. PET Clin..

[7] Vandenberghe S., Marsden P.K. (2015). PET-MRI: A review of challenges and solutions in the development of integrated multimodality imaging. Phys. Med. Biol..

[8] Bandettini P.A. (2012). Twenty years of functional MRI: The science and the stories. NeuroImage.

[9] Chen Y., An H. (2017). Attenuation Correction of PET/MR Imaging. Magn. Reson. Imaging Clin. N. Am..

[10] Catana C., Laforest R., An H., Boada F., Cao T., Faul D., Jakoby B., Jansen F.P., Kemp B.J., Kinahan P.E. (2020). Attenuation correction for human PET/MRI studies. Phys. Med. Biol..

[11] Sousa J.M., Appel L., Merida I., Heckemann R.A., Costes N., Engström M., Papadimitriou S., Nyholm D., Ahlström H., Hammers A. (2020). Accuracy and precision of zero-echo-time, single- and multi-atlas attenuation correction for dynamic [11C]PE2I PET-MR brain imaging. EJNMMI Phys..

[12] Izquierdo-Garcia D., Sawiak S.J., Knesaurek K., Narula J., Fuster V., Machac J., Fayad Z.A. (2014). Comparison of MR-based attenuation correction and CT-based attenuation correction of whole-body PET/MR imaging. Eur. J. Nucl. Med. Mol. Imaging.

[13] Yang X., Wang T., Lei Y., Higgins K., Liu T., Shim H., Curran W.J., Mao H., Nye J.A. (2018). MRI-based attenuation correction for brain PET/MRI based on anatomic signature and machine learning. Phys. Med. Biol..

[14] Ladefoged C.N., Hansen A.E., Henriksen O.M., Bruun F.J., Eikenes L., Øen S.K., Karlberg A., Højgaard L., Law I., Andersen F.L. (2020). AI-driven attenuation correction for brain PET/MRI: Clinical evaluation of a dementia cohort and importance of the training group size. NeuroImage.

[15] Del Guerra A., Ahmad S., Avram M., Belcari N., Berneking A., Biagi L., Bisogni M.G., Brandl F., Cabello J., Camarlinghi N. (2018). TRIMAGE: A dedicated trimodality (PET/MR/EEG) imaging tool for schizophrenia. Eur. Psychiatry.

[16] Neuner I., Rajkumar R., Brambilla C.R., Ramkiran S., Ruch A., Orth L., Farrher E., Mauler J., Wyss C., Kops E.R. (2019). Simultaneous PET-MR-EEG: Technology, Challenges and Application in Clinical Neuroscience. IEEE Trans. Radiat. Plasma Med. Sci..

[17] Belcari N., Bisogni M.G., Camarlinghi N., Carra P., Cerello P., Morrocchi M., Patera A., Sportelli G., Del Guerra A. (2019). Design and Detector Performance of the PET Component of the TRIMAGE PET/MR/EEG Scanner. IEEE Trans. Radiat. Plasma Med. Sci..

[18] Wienhard K., Schm M., Casey M.E., Baker K., Bao J., Eriksson L., Jones W.F., Knoess C., Lenox M., Lercher M. (2002). The ECAT HRRT: Performance and first clinical application of the new high resolution research tomograph. EEE Trans. Nucl. Sci..

[19] Yamaya T., Hagiwara N., Obi T., Yamaguchi M., Ohyama N., Kitamura K., Hasegawa T., Haneishi H., Yoshida E., Inadama N. (2005). Transaxial system models for jPET-D4 image reconstruction. Phys. Med. Biol..

[20] Yamaya T., Yoshida E., Kitamura K., Obi T., Tanimoto K., Yoshikawa K., Ito H., Murayama H. (2006). First human brain images of the jPET- D4 using 3D OS-EM with a pre-computed system matrix. Proceedings of the IEEE Nuclear Science Symposium Conference Record.

[21] Yamaya T., Yoshida E., Toramatsu C., Nishimura M., Shimada Y., Inadama N., Shibuya K., Nishikido F., Murayama H. (2009). Preliminary study on potential of the jPET-D4 human brain scanner for small animal imaging. Ann. Nucl. Med..

[22] Grogg K.S., Toole T., Ouyang J., Zhu X., Normandin M.D., Li Q., Johnson K., Alpert N.M., El Fakhri G. (2016). National Electrical Manufacturers Association and clinical evaluation of a novel brain PET/CT scanner. J. Nucl. Med..

[23] Yamamoto S., Honda M., Oohashi T., Shimizu K., Senda M. (2011). Development of a brain PET system, PET-Hat: A wearable PET system for brain research. IEEE Trans. Nucl. Sci..

[24] Moliner L., Rodríguez-Alvarez M.J., Catret J.V., González A., Ilisie V., Benlloch J.M. (2019). NEMA Performance Evaluation of CareMiBrain dedicated brain PET and Comparison with the whole-body and dedicated brain PET systems. Sci. Rep..

[25] Watanabe M., Saito A., Isobe T., Ote K., Yamada R., Moriya T., Omura T. (2017). Performance evaluation of a high-resolution brain PET scanner using four-layer MPPC DOI detectors. Phys. Med. Biol..

[26] Kolb A., Wehrl H.F., Hofmann M., Judenhofer M.S., Eriksson L., Ladebeck R., Lichy M.P., Byars L., Michel C., Schlemmer H.P. (2012). Technical performance evaluation of a human brain PET/MRI system. Eur. Radiol..

[27] González A.J., Majewski S., Sánchez F., Aussenhofer S., Aguilar A., Conde P., Hernández L., Vidal L.F., Pani R., Bettiol M. (2016). The MINDView brain PET detector, feasibility study based on SiPM arrays. Nucl. Instrum. Methods Phys. Res. A.

[28] Gonzalez A.J., Gonzalez-Montoro A., Vidal L.F., Barbera J., Aussenhofer S., Hernandez L., Moliner L., Sanchez F., Correcher C., Pincay E.J. (2019). Initial Results of the MINDView PET Insert Inside the 3T mMR. IEEE Trans. Radiat. Plasma Med Sci..

[29] Jung J.H., Choi Y., Jung J., Kim S., Lim H.K., Im K.C., Oh C.H., Park H.W., Kim K.M., Kim J.G. (2015). Development of PET/MRI with insertable PET for simultaneous PET and MR imaging of human brain. Med. Phys..

[30] Nishikido F., Fujiwara M., Tashima H., Akram M.S.H., Suga M., Obata T., Yamaya T. (2017). Development of a full-ring ‘‘add-on PET’’ prototype: A head coil with DOI-PET detectors for integrated PET/MRI. Nucl. Instrum. Methods Phys. Res. A.

[31] Nishikido F., Obata T., Shimizu K., Suga M., Inadama N., Tachibana A., Yoshida E., Ito H., Yamaya T. (2014). Feasibility of a brain-dedicated PET-MRI system using four-layer DOI detectors integrated with an RF head coil. Nucl. Instrum. Methods Phys. Res. A.

[32] Tsuda T., Murayama H., Kitamura K., Yamaya T., Yoshida E., Omura T., Kawai H., Inadama N., Orita N. (2004). A four-layer depth of interaction detector block for small animal PET. IEEE Trans. Nucl. Sci..

[33] Won J.Y., Park H., Lee S., Son J.W., Chung Y., Ko G.B., Kim K.Y., Song J., Seo S., Ryu Y. (2021). Development and Initial Results of a Brain PET Insert for Simultaneous 7-Tesla PET/MRI Using an FPGA-Only Signal Digitization Method. IEEE Trans. Med. Imaging.

[34] Karp J.S., Daube-Witherspoon M.E., Hoffman E.J., Lewellen T.K., Links J.M., Wong W.H., Hichwa R.D., Casey M.E., Colsher J.G., Hitchens R.E. (1991). Performance standards in positron emission tomography. J. Nucl. Med..

[35] NEMA NU 2-2001, National Electrical Manufacturers Association (2001). NEMA Standards Publication NU 2-2001: Performance Measurements of Positron Emission Tomographs.

[36] NEMA NU 2-2017, National Electrical Manufacturers Association (2007). NEMA Standards Publication NU 2-2007. Performance Measurements of Positron Emission Tomographs.

[37] NEMA NU 2-2018, National Electrical Manufacturers Association (2018). NEMA Standards Publication NU 2-2018. Performance Measurements of Positron Emission Tomographs.

[38] Salleh A., Fleury J., de la Taille C., Seguin-Moreau N., Dulucq F., Martin-Chassard G., Callier S., Thienpont D., Raux L. (2015). Triroc: A Multi-Channel SiPM Read-Out ASIC for PET/PET-ToF Application. IEEE Trans. Nucl. Sci..

[39] Sportelli G., Ahmad S., Belcari N., Bisogni M.G., Camarlinghi N., Di Pasquale A., Dussoni S., Fleury J., Morrocchi M., Zaccaro E. (2017). The TRIMAGE PET Data Acquisition System: Initial Results. IEEE Trans. Radiat. Plasma Med. Sci..

[40] Reader A.J., Julyan P.J., Williams H., Hastings D.L., Zweit J. (2003). EM algorithm system modeling by image-space techniques for PET reconstruction. IEEE Trans. Nucl. Sci..

[41] Fazendeiro L., Ferreira N.C., Blanco A., Fonte P. (2004). EM reconstruction algorithm with resolution modeling applied to an RPC-PET prototype. IEEE Symp. Conf. Rec. Nucl. Sci..

[42] Varrone A., Sjöholm N., Eriksson L., Gulyás B., Halldin C., Farde L. (2009). Advancement in PET quantification using 3D-OP-OSEM point spread function reconstruction with the HRRT. Eur. J. Nucl. Med. Mol. Imaging.

[43] Wang G., Qi J. (2012). Penalized likelihood PET image reconstruction using patch-based edge-preserving regularization. IEEE Trans. Med. Imaging.

[44] NEMA NU 2-2012 (2012). Performance Measurements of Positron Emission Tomographs.

[45] NEMA NU 4-2008 (2008). Performance Measurements of Small Animal Positron Emission Tomographs.

[46] Jan S., Santin G., Strul D., Staelens S., Assie K., Autret D., Avner S., Barbier R., Bardies M., Bloomfield P.M. (2004). GATE: A simulation toolkit for PET and SPECT. Phys. Med. Biol..

[47] Gong K., Cherry S., Qi J. (2016). On the Assessment of Spatial Resolution of PET Systems with Iterative Image Reconstruction. Phys. Med. Biol..

